# Mechanical Performance of Extensive Restorations Made with Short Fiber-Reinforced Composites without Coverage: A Systematic Review of In Vitro Studies

**DOI:** 10.3390/polym16050590

**Published:** 2024-02-21

**Authors:** András Jakab, Dániel Palkovics, Veronika T. Szabó, Balázs Szabó, Eszter Vincze-Bandi, Gábor Braunitzer, Lippo Lassila, Pekka Vallittu, Sufyan Garoushi, Márk Fráter

**Affiliations:** 1 Department of Operative and Esthetic Dentistry, Faculty of Dentistry, University of Szeged, H-6720 Szeged, Hungary; jakab.andras.gabor@gmail.com (A.J.); tszaboveronika@gmail.com (V.T.S.); 2 Department of Periodontology, Faculty of Dentistry, Semmelweis University, H-1088 Budapest, Hungary; dpalkovics@gmail.com; 3 Department of Periodontology, Faculty of Dentistry, University of Szeged, H-6720 Szeged, Hungary; drszabobalazs77@gmail.com; 4 Dr. Borbáth Dental and Implantology Center, H-6800 Hódmezővásárhely, Hungary; vbeszter96@gmail.com; 5 dicomLAB Dental Ltd., H-6726 Szeged, Hungary; braunitzergabor@gmail.com; 6 Department of Biomaterials Science and Turku Clinical Biomaterials Center—TCBC, Institute of Dentistry, University of Turku, FI-20520 Turku, Finland; liplas@utu.fi (L.L.); pekval@utu.fi (P.V.); sufgar@utu.fi (S.G.)

**Keywords:** fiber reinforcement, short fibers, short-fiber-reinforced composite, fracture toughness, fracture pattern, load-bearing capacity

## Abstract

In recent years, composite resin materials have been the most frequently used materials for direct restorations of posterior teeth. These materials have some clinically relevant limitations due to their lack of fracture toughness, especially when used in larger cavities with high volume factors or when utilized as direct or indirect overlays or crown restorations. Recently, short-fiber-reinforced composite materials have been used in bi-structure restorations as a dentine substituting material due to their superior mechanical properties; however, there is no scientific consensus as to whether they can be used as full restorations. The aim of our review was to examine the available literature and gather scientific evidence on this matter. Two independent authors performed a thorough literature search using PubMed and ScienceDirect up until December 2023. This study followed the PRISMA guidelines, and the risk of bias was assessed using the QUIN tool. The authors selected in vitro studies that used short-fiber-reinforced composite materials as complete restorations, with a conventional composite material as a comparison group. Out of 2079 potentially relevant articles, 16 met our inclusion criteria. All of the included studies reported that the usage of short-fiber-reinforced composites improved the restoration’s load-bearing capacity. Fifteen of the included publications examined the fracture pattern, and thirteen of them reported a more favorable fracture outcome for the short-fiber-reinforced group. Only one article reported a more favorable fracture pattern for the control group; however, the difference between groups was not significant. Within the limitations of this review, the evidence suggests that short-fiber-reinforced composites can be used effectively as complete restorations to reinforce structurally compromised teeth.

## 1. Introduction

With the development of adhesive techniques, clinicians are routinely using composite fillings in both the anterior and posterior regions [1,2]. Modern restorations are expected to not only restore the aesthetics and function of teeth, but also to strengthen the remaining tooth material, protecting against fractures [3]. Excessive cavity preparations and root canal treatments make the teeth more prone to fractures [4,5]. The more dentine that is lost due to caries, previous restorations, or fractures, the less resistant teeth become to fracture. The loss of a single marginal ridge can lead to a 46% loss in tooth rigidity in premolars, and in the case of a mesio-occluso-distal (MOD) cavity, this ratio can be as high as 63% [6]. Besides the absence of these structurally important elements, the depth of the cavity plays a significant role in the mechanical integrity of the teeth. The volume factor, which refers to the size of the missing tooth material, is a factor with key importance regarding the weakening effect of the lesion [7,8]. It has been shown that while a shallower cavity (referring to minimal hard tissue loss) can be safely restored with a direct composite restoration [9], more extensive restorations made with such restorative techniques exhibit significantly lower fracture resistance [10].

In recent years, composite resin materials have become the most frequently used materials for direct restorations of posterior teeth. However, due to their physical limitations, clinical failure may occur in the case of extensive restorations. The two most concerning problems with conventional particulate filler composites (PFCs) are polymerization shrinkage [11,12,13,14] and that—even though they are brittle, strong materials—they lack fracture toughness [15]. The problem of low fracture toughness is particularly evident in larger direct restorations, as the volume of the material increases. Fracture toughness is a mechanical property that determines how well brittle materials resist the catastrophic propagation of flaws under an applied load [16,17,18]. This parameter is considered to be the measure of fatigue resistance, which predicts structural performance [19].

Short-fiber-reinforced composite (SFRC) materials seem to offer a solution to this problem, as these materials are much closer to dentine in terms of their physical parameters [16]. In previous studies, it has been shown that even deep MOD cavities can be reinforced by the application of SFRCs in direct restorations [15,20]. Due to this reinforcing effect, in recent years, numerous innovative restoration techniques and materials have appeared. The use of SFRCs has expanded the possible applications of direct extensive restorations both in vital and root-canal-treated teeth. For structurally compromised teeth, an indirect restoration was usually performed in the past; however, new materials and techniques may allow clinicians to perform a less time-consuming and more cost-effective extensive direct restoration in these cases (such as direct crowns or overlays). The question arises if maximizing the SFRC content in restorations could maximize the potential reinforcing effect in direct and indirect situations. Our study aims to collect and evaluate the available evidence on this subject.

## 2. Materials and Methods

This systematic review was carried out according to the Preferred Reporting Items for Systematic Review and Meta-Analysis guidelines (PRISMA) [21].

### 2.1. Search Strategy

Sources: A review of the literature was conducted using PubMed and ScienceDirect until December 2023. Articles were gathered using keywords such as “fiber reinforcement”, “fiber-reinforced composite”, “fiber-reinforced restoration”, “SFRC”, and “EverX”. While our emphasis was on articles from the current decade, a few older publications (n = 4) were incorporated due to their high relevance. Boolean operators (“AND” and “OR”) were employed to connect the keywords, and these terms were adjusted accordingly for each database. Table 1 shows the used search strategies.

### 2.2. Study Screening and Selection

The collected articles were organized in a reference management software (Mendeley Reference Manager 2.106.0). After scanning for duplicates, two independent examiners (A.J. and M.F.) carefully reviewed the title and the abstract of the articles. A title was discarded if both examiners agreed it was irrelevant. Eligible abstracts were selected for a full-text review.

### 2.3. Eligibility Criteria

Eligible studies were in vitro studies that used short-fiber-reinforced composite materials as complete restorations, compared to a control group restored with conventional particulate filler composite materials. Only studies testing load-bearing capacity were included. Further criteria for eligibility encompassed studies published in peer-reviewed journals and written in English. These studies were required to incorporate the search terms within either the title or the abstract.

### 2.4. Data Synthesis

The studies meeting the inclusion criteria underwent thorough examination, with relevant information systematically gathered in a Microsoft Excel document. For each included publication, authors’ names, article title, publication year, experimental and control groups, type of short fiber employed, restoration type, as well as the primary outcomes and conclusions were documented.

### 2.5. Quality Assessment

The risk of bias was determined for each included publication using the Quality Assessment Tool for In Vitro Studies (QUIN), which consists of 12 criteria [22]. Each criterion is scored from 0 to 2 (0: not specified; 1: inadequately specified; 2: adequately specified; NA: not applicable). The final score is determined by the following formula: final score = (total score × 100)/(2 × number of criteria applicable). According to the final score, the article is graded as having low, medium, or high risk of bias (>70%, low risk of bias; 50–70%, medium risk of bias; <50%, high risk of bias).

## 3. Results

**Figure 1:**
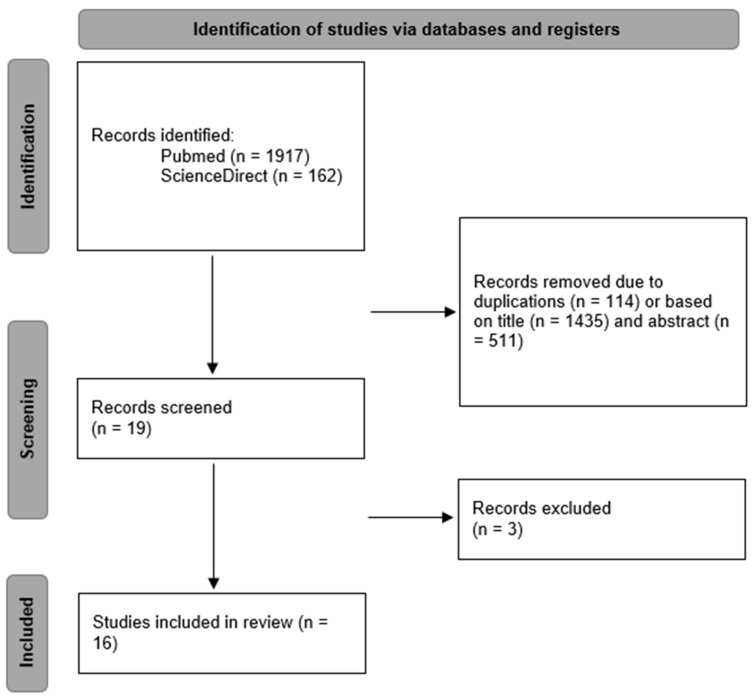
PRISMA flow diagram of the screening and selection process.

After the application of our search strategy, 2079 records were identified. After the removal of the duplicates (n = 114), 1995 articles were submitted to title and abstract screening. After screening, 19 articles were examined on the full-text level. After the removal of articles that did not meet our inclusion criteria (n = 3), 16 articles were included in the final review. Figure 1 shows the screening and selection process in a PRISMA flow diagram [21].

Four of the sixteen studies used extracted human teeth [23,24,25,26], while twelve studies used artificial plastic [27,28,29], zirconia [30,31,32,33,34,35], or cobalt-chromium [36,37,38] models as a base for the restorations. All included studies conducted mechanical testing to measure the load-bearing capacity of the restorations. After loading, they examined the fracture patterns, too. Three of the included publications involved further material investigations and measured some of the physical parameters of the tested materials, such as fracture toughness [27,34,37]. In accordance with our inclusion criteria, all the selected studies tested restorations solely made with an SFRC material and included a comparison group made with conventional PFC. Ten of the selected studies used an SFRC with a paste consistency (experimental fiber composite, EverX Posterior) [23,24,25,29,30,31,32,33,34,35], six of them used a flowable SFRC [26,27,28,36,37,38], and four of them made restorations using a short-fiber-reinforced CAD-CAM composite material [30,32,36,37]. All the included studies reported significantly higher load-bearing capacities for the groups restored solely with SFRCs compared to the PFC control groups. In one of the selected publications, where the authors tested both a paste and a CAD-CAM SFRC material for inlay-retained fixed partial dentures (IRFPDs), the PFC group performed slightly better than the paste-consistency SFRC group [30]. However, in this case, the CAD-CAM SFRC material performed significantly better than all the other tested groups in terms of load-bearing capacity. In another publication, the authors tested the materials both with and without a conventional fiber-reinforced composite (FRC) post in the restoration [29]. In this case, the SFRC group without the FRC post outperformed the control group. However, when a conventional FRC post was applied in the restoration, the PFC group performed better. Furthermore, in those articles where a material analysis was performed, in all cases, significantly higher fracture toughness was measured in the SFRC groups. Fifteen of the included studies examined the fracture pattern in the failed specimens [23,24,25,26,28,29,30,31,32,33,34,35,36,38]. In thirteen cases, a more favorable fracture pattern was reported for the SFRC groups compared to their controls. Bielic et al. found that both the SFRC and the control groups had favorable fracture patterns [29]. In one case, the PFC group showed a slightly more favorable pattern, but the difference was statistically not significant [24]. Table 2 summarizes the details of the included publications.

### 3.1. Quality Assessment of the Included In Vitro Trials

The details of the methodological quality of the publications are shown in Table 3. Thirteen of the publications had medium risk of bias, while three of the studies had low risk [24,26,38].

### 3.2. Quantitative Assessment of the Included Publications

The highest median value for LBC was measured in a group restored by flowable SFRC (EverX Flow) onlays at 3990 N (±330 N) [28]. The highest fracture toughness was exhibited by an FRC CAD-CAM material at 2.9 MPa/m^2^ [37], and the lowest was 1.0 MPa/m^2^, which was measured for a conventional composite filling material [34]. Table 4 summarizes the methodology of the mechanical tests applied in the assessed studies, while Table 5 summarizes the load-bearing results from the assessed studies.

## 4. Discussion

Fiber reinforcement for restoring structurally compromised teeth is a frequently discussed subject. Restorations after endodontic treatments, large decays, and replacement of large fillings are part of the everyday dental routine, and there is a constant need for the reinforcement of these teeth. Although PFC materials have some limitations in terms of their physical properties, they are routinely used to restore large MOD cavities in molars and premolars [39,40]. One approach to tackling the issue of polymerization shrinkage is layering the material while considering the variable bondability across various dental tissues. With the “decoupling with time” concept, clinicians can counter the stresses caused by the material’s shrinkage during and after polymerization [40]. A generally accepted biomimetic restorative approach suggests replacing enamel with feldspathic porcelain or glass ceramic and dentine with hybrid composites [41]. Even though this technique shows promising results in the case of performing indirect restorations, it does not take ideal fracture toughness into consideration, and the lack of toughness can lead to catastrophic fractures. Although certain mechanical properties of conventional composites, like flexural strength, flexural modulus, and thermal expansion coefficient, may resemble those of dentine from a biomimetic perspective, the material’s microstructure significantly differs from that of dentine [41]. Conventional PFC materials consist of filler particles embedded in a resin matrix, while dentine consists of collagen fibers in a hydroxyapatite matrix. As a result, PFC materials are rigid, brittle materials and they are incapable of stopping crack propagation under loading, which can lead to irreparable, catastrophic fractures [16]. For the above-mentioned reasons, direct composite restorations might not be the ideal solution to higher-volume-factor cavities. Fiber reinforcement in composite restorations can strengthen the compromised tooth and the restoration. These fibers can make the composite’s microstructure more dentine-like, as they provide the material a much higher fracture toughness than PFC; thus, they are more suitable for restoring missing dentine. The volume of the material, the size and the orientation of the fibers, and the fibers’ adhesion to the matrix can all influence the mechanical performance of the material. The transfer of stress from the polymer matrix to the fibers greatly affects the material’s mechanical performance [42]. Also, critical fiber length, which is calculated from the diameter from the fibers, is of paramount importance. For advanced fiber-reinforced composites, the length of the fibers should be as much as 50 times the diameter of the fiber [16]. In the past, there have been two low-aspect-ratio packable SFRC materials (Alert, Pentron; Nulite F, NSI Dental) available on the market. Although they exhibited acceptable mechanical performance, some clinical issues, including surface roughness and wear resistance, as well as material and cusp fractures, hindered their widespread adoption as a general dentine substitute [43,44]. Alert has a fiber length of 60–80 μm, with a 6–10-micrometer diameter, and Nulite F has a fiber length of 150–200 μm with a 9 μm diameter. In both cases, the fibers are well below the critical fiber length, which explains the above-mentioned problems [42]. This is also in accordance with the difference in fracture toughness between these earlier SFRCs and the more advanced, modern materials. The fracture toughness of Alert is measured at 1.7 MPa/m^2^, while that of EverX Flow is measured at 2.8 MPa/m^2^ [37]. In modern SFRC materials, the fibers are randomly oriented, which means they are capable of strengthening the material in every direction. Longer, conventional, unidirectional fiber-containing posts often deliver a stronger reinforcement, yet achieving a precise orientation can pose practical challenges. Nowadays, SFRC materials are often used to replace dentine because they are easy to use and provide a quick solution to the restoration of heavily compromised teeth [20,45]. Excessive wear and surface roughness problems associated with earlier SFRC materials appear to be resolved. EverX Flow had significantly lower wear value after 15,000 chewing simulation cycles than conventional PFCs, and it did not exhibit a coarse surface after the test. This SFRC material fulfilled the American Dental Association’s criterion for wear [46]. In an earlier study, after 100,000 cycles of brushing simulation, it was found that SFRC had a similar wear and surface roughness to that of PFC [47]. During the polishing of these materials, the fibers on the surface suffer microfractures; thus, they can be polished together with the resin matrix [46]. As a future perspective, with the advancement of these SFRC materials, it might be possible to use them not just as a dentine substitute, but rather for complete restorations. A usual concern regarding these materials is their water absorption after restoration. Lassila et al. compared the water absorption of flowable SFRC to conventional bulk-fill PFC. After 36 days, the material’s water absorption was the second best (0.5 wt%), while other regular bulk-fill composite materials had much higher values (Estelite Bulk Fill Flow 1.1 wt%) [46]. Regarding fracture patterns, SFRCs show a promising performance in stopping and deflecting crack formation [48,49,50]. As it is the fracture pattern that will determine the restorability of teeth in case of a fracture, it is of high importance. According to Scotti and co-workers, a distinction can be made between restorable or non-restorable fractures based on optical microscopic observation (preferably with a two-examiner agreement). A restorable fracture is above the cemento-enamel junction (CEJ), meaning that in case of fracture, the tooth can be restored, while a non-restorable fracture extends below the CEJ and the tooth will likely need to be extracted [51]. The energy-absorbing and stress-distributing fibers allow crack propagation to be deflected toward the peripheries of the material, which can result in a much more favorable fracture pattern.

For the above reasons, this review aimed to gather information on whether the maximization of SFRCs can further strengthen restorations and if their use contributes to a more favorable fracture pattern.

### 4.1. Fracture Resistance

Fracture resistance is one of the most important characteristics of materials used in posterior region as it determines the longevity of the restorations. All the included studies tested the restorations’ load-bearing capacity. In clinical situations, the load-bearing capacity is highly dependent on the quantity and quality of the remaining tooth structure. Four of the included studies used extracted human teeth for these tests [23,24,25,26]. While these in vitro investigations have a high clinical relevance, the usage of human teeth adds another variable to this equation in terms of mechanical parameters and survival of the restoration–tooth unit. In these cases, the measured load-bearing capacity values came out much lower than in the rest of the included publications, where they used standardized, artificial models.

In all but one of the studies, the uncovered SFRC group performed significantly better than the control PFC group. Bijelic et al. did not compare the SFRC and the control groups directly [29]. Even though the SFRC group performed better as an anterior crown restoration without the application of an FRC post (238 ± 43 N), with the insertion of a post, the PFC group performed slightly better (265 ± 59 N). The authors did not report a significant difference between the groups [29]. Apart from this study, all the other articles reported a significantly higher fracture resistance for the SFRC group compared to the PFC control group. Eight of the included studies compared an uncovered, plain SFRC group to a bi-layered SFRC + PFC group as well [25,26,28,31,34,35,38]. In all eight publications, the uncovered SFRC performed better than the bi-layered group. Lassila et al. compared EverX Flow covered by 2 mm PFC on the occlusal surface to uncovered flowable SFRC in posterior crown restorations. They found that the uncovered group (3866 ± 263 N) performed significantly better (*p* < 0.05) than the covered ones (2000–2250 N) [27]. In another publication, they compared posterior crown restorations made with a flowable SFRC (EverX Flow) core covered by PFC layers of different thicknesses (0.5, 1, 1.5, 2). They also included an uncovered, plain SFRC group and a fully PFC-made group (1907.7 ± 179 N). Regression analysis showed that by decreasing the thickness of the PFC layer, the load-bearing capacity increased. The uncovered, plain SFRC group had a significantly higher load-bearing capacity than all the tested groups (3990 ± 330 N) [28]. Garoushi et al. tested direct onlay restorations made of plain PFC, SFRC + PFC, and plain SFRC [35]. Similarly to the crown restorations, the uncovered SFRC group exhibited the highest load-bearing capacity, which was significantly higher (1733 N) than that of the control PFC group (1081 N).

### 4.2. Fracture Pattern

Structurally compromised teeth are prone to fracture, mainly due to the high amount of lost tooth material, the presence of cracks, and the loss of structurally important parts, such as marginal ridges [52,53]. If a fracture occurs, the fracture pattern will determine the restorability of the tooth, and for that reason, it is of high importance.

Fifteen of the included articles investigated fracture patterns. Thirteen reported a favorable pattern for the SFRC groups [23,25,26,28,30,31,32,33,34,35,36,38]. Cekic-Nagas et al. tested both an SFRC paste composite (EverX Posterior) and a CAD-CAM SFRC material [30]. They found that while EverX Posterior did not perform significantly better than the control group, the CAD-CAM material showed a significantly more favorable fracture pattern than the rest of the groups. This could be explained by the amount of fibers in the material. The CAD-CAM version is more similar in terms of type of fibers to the flowable version of EverX, which means that because of the shorter fibers, the material has a higher fiber ratio. Bielic et al. found that both the SFRC and the control group had favorable fracture patterns [29]. In another publication, they reported that the control group showed a slightly more favorable fracture pattern, but the difference from the SFRC group was not statistically significant [24].

Despite the manufacturer’s recommendation to use SFRCs exclusively as a substitute for missing dentin in direct and indirect restorations while avoiding contact with the oral environment, the study’s findings suggest that employing SFRCs without surface coverage in various simulated clinical scenarios yielded satisfactory outcomes in terms of restoration fracture behavior. This might improve the clinical performance of extensive direct composite restorations; however, it is necessary to conduct clinical trials to obtain more reliable and conclusive findings.

## 5. Conclusions

Within the limitations of this review, in vitro evidence suggests that short-fiber-reinforced composite materials effectively reinforce structurally compromised teeth. These materials exhibit higher fracture toughness, akin to dentine, imparting greater resilience, and the restorations created with SFRCs demonstrate heightened load-bearing capacities. Regarding fracture patterns, most publications documented more favorable outcomes when using SFRCs. Some studies compared groups using SFRCs with and without a PFC covering, and in these instances, the uncovered SFRC groups demonstrated superior performance. For these reasons and due to advancements in areas such as two-body wear and surface roughness, SFRCs may hold promise for future clinical use as complete restorations; however, further research is warranted in this area.

## Figures and Tables

**Table 1:** Search strategy in each database.

Database	Search Strategy
Pubmed	((((Fiber reinforcement [Title/Abstract]) OR (fiber-reinforced composite [Title/Abstract])) OR (fiber-reinforced restoration [Title/Abstract])) OR (SFRC [Title/Abstract])) OR (EverX [Title/Abstract])
ScienceDirect	in title/abstract: (“tooth” OR “teeth”) AND (“Fiber reinforcement” OR “fiber-reinforced composite” OR “fiber-reinforced restoration” OR “sfrc” OR “everx”)

**Table 2:** Details of the included publications.

First Author	Type of SFRC	Type of Restoration	Type of Mechanical Testing	Tested Parameters	Main Conclusion
L. Lassila [27]	EverX Flow	Posterior crown	Statical loading	LBC + FT + FP	“Restorations combining a fiber-reinforced composite core and a surface layer of conventional composite, displayed promising performance related to fracture-behavior.”
I. Cekic-Nagas [30]	EverX Posterior, SFRC CAD-CAM	IRFPD	Statical loading	LBC + FP	“FRC had significantly affected load-bearing capacity of tested inlay-retained FDPs”
L. Lassila [36]	EverX Flow, SFRC CAD-CAM	IRFDP	Dynamic and statical loading	LBC + FP	“CAD/CAM-fabricated IRFPDs made of experimental SFRC blocks have shown promising performance in clinical testing in terms of fracture behavior”.
S. Garoushi [23]	Experimental FRC	Anterior crown	Statical loading	LBC + FP	“Short glass fiber reinforced semi-IPN composite resin demonstrated improved load-bearing capacity compared with conventional particulate filler restorative composite resin”.
L. Lassila [28]	EverX Flow	Posterior crown	Statical loading	LBC + FP	“Restorations combining a thick FRC-core and a thin surface layer of PFC (0.5–1 mm), displayed promising performance related to fracture-behavior and load-bearing capacity”.
E. Mangoush [37]	EverX Flow, SFRC CAD-CAM	Anterior crown	Statical loading	LBC + FT + FM	“Within the limitation of this study, single-structure CAD/CAM fabricated restorations made of experimental SFRC blocks displayed promising performance related to fracture-behavior”.
S. Garoushi [31]	Experimental FRC	FDP	Statical loading	LBC + FP	“Short glass fiber reinforced semi-IPN composite resin revealed improvement in load-bearing capacity compared with the conventional particulate filler veneering composite”.
K. Nagata [32]	EverX Posterior, SFRC CAD-CAM	Anterior crown	Statical loading	LBC + FP	“Single-structure FRC restorations showed higher fracture resistance than the restorations made from PFC, leucite-reinforced ceramic, and lithium disilicate”.
J. Bijelic [29]	Experimental FRC	Anterior crown	Statical loading	LBC + FP	“…individually formed fiber-reinforced (FRC) root canal post improved the fracture load of the post-crown system and significantly contributed to the reinforcement and strengthening the restored teeth”.
S. Garoushi [35]	EverX Posterior	Onlay	Statical loading	LBC + FP	“Onlay restorations combining base of short fiber reinforced composite resin as substructure and surface layer of conventional composite resin displayed promising performance in high load-bearing areas”.
L. Lassila [38]	EverX Flow	Anterior crown	Statical loading	LBC + FP	“Using SFC as core material with conventional PFC veneering composite to strengthen anterior crown restoration proved to be a promising strategy for further testing”.
J. Bijelic [24]	Experimental FRC	Anterior crown	Statical loading	LBC + FP	“...the use of SFC as a restorative material of choice for fabricating the direct composite post-core-crown restorations of severely damaged incisors provided an improved load-bearing capacity greater than CC…”
F. Keulemans [33]	Experimental FRC	Inlay-retained FDP	Statical loading	LBC + FP	“…S-FRC seems to be a viable material for improving the framework of FRC-FDPs...”
J. Bijelic [34]	EverX Posterior	Anterior crown	Dynamic and statical loading	LBC + FT + FM + FP	“SFC crowns showed good performance under static and fatigue loading. FT was the only in vitro test method that filtered as a clinically relevant parameter”.
S. Garoushi [25]	EverX Posterior	Class II filling and onlay	Statical loading	MGF + LBC + FP	“Based on the microleakage and compressive loading tests, base of short FRC resin and surface layer of conventional composite resin is the best combination”.
S. Garoushi [26]	EverX Flow	Overlay	Dynamic and statical loading	LBC + FP	“The most effective method for restoring large MOD cavities was found to be direct restoration using SFC either alone or as a bulk core in combination with PFC composite”.

LBC: load-bearing capacity; FT: fracture toughness; FP: fracture pattern; FM: flexural modulus; MGF: marginal gap formation; FDP: fixed dental prosthesis.

**Table 3:** Quality assessment of the in vitro studies using the QUIN tool.

First Author	Clearly Stated Aims/Objectives	Sample Size Calculation	Explanation of Sampling Technique	Details of Control Group	Explanation of Methodology	Operator Details	Randomization	Method of Measurement of Outcome	Outcome Assessor Details	Blinding	Statistical Analysis	Presentation of Results	Risk of Bias
L. Lassila [27]	2	0	2	2	2	0	NA	2	0	NA	2	2	Medium
I. Cekic-Nagas [30]	2	0	2	1	2	0	NA	2	0	NA	2	2	Medium
L. Lassila [36]	2	0	2	1	2	0	NA	2	0	NA	2	2	Medium
S. Garoushi [23]	2	0	2	2	2	0	NA	2	0	NA	2	2	Medium
L. Lassila [28]	2	0	2	2	2	0	NA	2	0	NA	2	2	Medium
E. Mangoush [37]	2	0	2	1	2	0	NA	2	0	NA	2	2	Medium
S. Garoushi [31]	2	0	2	2	2	0	NA	2	0	NA	2	2	Medium
K. Nagata [32]	2	0	2	1	2	0	NA	2	0	NA	2	2	Medium
J. Bijelic [29]	2	0	2	2	2	0	NA	2	0	NA	2	2	Medium
S. Garoushi [35]	2	0	2	2	2	0	NA	2	0	NA	2	2	Medium
L. Lassila [38]	2	0	2	1	2	2	NA	2	1	NA	2	2	Low
J. Bijelic [24]	2	0	2	2	2	1	NA	2	1	NA	2	2	Low
F. Keulemans [33]	2	0	2	1	2	0	NA	2	0	NA	2	2	Medium
J. Bijelic [34]	2	0	2	2	2	0	NA	2	0	NA	2	2	Medium
S. Garoushi [25]	2	0	2	2	2	0	NA	2	0	NA	2	2	Medium
S. Garoushi [26]	2	0	2	2	2	2	NA	2	2	NA	2	2	Low

0–2 (0: not specified; 1: inadequately specified; 2: adequately specified; NA: not applicable). Final score = (total score × 100)/(2 × number of criteria applicable). According to the final score the article is graded low, medium, or high risk of bias (>70%, low risk of bias; 50–70%, medium risk of bias; <50%, high risk of bias).

**Table 4:** Methodology of the mechanical tests performed in the included articles.

First Author	Statical Loading			Dynamical Loading
	Direction of Loading	Crosshead Speed	Loading Tip Diameter	
L. Lassila [27]	Vertical (long axis)	1 mm/min	5 mm	No
I. Cekic-Nagas [30]	Vertical (long axis)	1 mm/min	6 mm	No
L. Lassila [36]	Vertical (long axis)	1 mm/min	NA	10,000 cycles, Fmax = 500 N for 20 s, 1.2 Hz
S. Garoushi [23]	45° Oblique	1 mm/min	2 mm	No
L. Lassila [28]	Vertical (long axis)	1 mm/min	5 mm	No
E. Mangoush [37]	45° Oblique	1 mm/min	NA	No
S. Garoushi [31]	Vertical (long axis)	1 mm/min	6 mm	No
K. Nagata [32]	45° Oblique	1 mm/min	NA	No
J. Bijelic [29]	45° Oblique	1 mm/min	NA	No
S. Garoushi [35]	Vertical (long axis)	1 mm/min	3 and 6 mm	No
L. Lassila [38]	45° Oblique	1 mm/min	NA	No
J. Bijelic [24]	45° Oblique	1 mm/min	NA	No
F. Keulemans [33]	Vertical (long axis) and Oblique (buccal cusp)	1 mm/min	6 mm	No
J. Bijelic [34]	45° Oblique	1 mm/min	NA	Dynamic: 10,000 cycles, 1.0 Hz
S. Garoushi [25]	Vertical (long axis)	1 mm/min	5 mm	No
S. Garoushi [26]	Vertical (long axis)	1 mm/min	5 mm	500,000 cycles, Fmax = 150 N, 1.5 Hz

NA: not available

**Table 5:** Load-bearing capacity test results.

First Author	LBC SFRC Group	LBC Control Group	Significance
L. Lassila [27]	3866 ± 263 N	1750–2250 N	*p* < 0.05
I. Cekic-Nagas [30]	CAD-CAM FRC: 896.1 ± 30.3 N	476.9 ± 20.3 N	*p* < 0.05
L. Lassila [36]	Paste SFRC: 476.9 ± 20.3 N		-
S. Garoushi [23]	Before aging SFRC CAD-CAM: 2624 ± 463 N	1427 ± 409 N	*p* < 0.05
L. Lassila [28]	Before aging SFRC Flow: 2521 ± 371 N		*p* < 0.05
E. Mangoush [37]	After aging SFRC CAD-CAM: 2775 ± 297 N	1599 ± 397 N	*p* < 0.05
S. Garoushi [31]	After aging SFRC Flow: 2404 ± 357 N		*p* < 0.05
K. Nagata [32]	349 N	173 N	*p* < 0.05
J. Bijelic [29]	3990 ± 330 N	1098 ± 179 N	*p* < 0.05
S. Garoushi [35]	SFRC CAD-CAM: 1650 ± 230 N	850–950 N	*p* < 0.05
L. Lassila [38]	SFRC Flow: 1310 ± 397 N		*p* < 0.05
J. Bijelic [24]	2171 N	1482 N	*p* < 0.05
F. Keulemans [33]	Paste SFRC: 1145 ± 89.6	580 ± 40 N	*p* < 0.05
J. Bijelic [34]	SFRC CAD-CAM: 913.6 ± 86.3		*p* < 0.05
S. Garoushi [25]	Without post: 238 N	Without post: 158 N	
	With post: 199 N	With post: 265 N	
L. Lassila [27]	1733 N	1081 N	*p* < 0.05
I. Cekic-Nagas [30]	~1700 N	~900 N	*p* < 0.05
L. Lassila [36]	515.8 ± 241.6 N	164.8 ± 95.1 N	*p* < 0.05
S. Garoushi [23]	Central fossa: 800 N	Central fossa: 702 ± 86 N	
L. Lassila [28]	Buccal cusp: 643 ± 8 N	Buccal cusp: 403 ± 62 N	*p* < 0.05
E. Mangoush [37]	954 ± 121 N	415 ± 75 N	*p* < 0.05
S. Garoushi [31]	1528 N	~900 N	*p* < 0.05
K. Nagata [32]	2674 ± 465 N	~1800 N	*p* < 0.05

## Data Availability

Not applicable.
